# Central Nervous System Disease Progression Among Patients With Metastatic Urothelial Carcinoma Treated With Enfortumab Vedotin: A Case Series

**DOI:** 10.1016/j.clgc.2023.11.014

**Published:** 2023-11-25

**Authors:** Chase Shipp, Tanya Jindal, Jonathan Chou, Terence W. Friedlander, Vadim S. Koshkin, Vipul Kumar

**Affiliations:** University of California San Francisco, Helen Diller Family Comprehensive Cancer Center, San Francisco, CA

## Introduction

Central nervous system (CNS) metastases are associated with significant morbidity and mortality and may arise from any systemic malignancy.^1^ CNS metastases are rare in urothelial cancer and have historically been reported in 1% to 3% of patients.^2–5^ In contrast to many other epithelial-based tumors such as melanoma, breast and lung, routine CNS imaging is not recommended in bladder cancer. ^6^ Patient outcomes after CNS metastasis in bladder cancer are generally poor, with an estimated median overall survival (mOS) of 3 months.^7^

The treatment landscape of metastatic urothelial cancer (mUC) has undergone dramatic changes in recent years with approvals of targeted agents, including antibody-drug conjugates (ADCs).^8^ Enfortumab vedotin (EV), directed against Nectin-4, was the first ADC approved by the FDA for treatment-refractory mUC in 2019.^9^ Recently, EV was approved in combination with pembrolizumab as frontline treatment for cisplatin-ineligible patients.^10^

Clinical trials of EV excluded patients with active CNS disease; consequently, pharmacokinetic and pharmacodynamic data for this drug in the CNS, as well as significant clinical experience in EV-treated patients with CNS involvement, are lacking.^9,11^ A single case series documented 3 CNS responses after EV treatment, suggesting potential activity of EV in the CNS.^12^ Other ADCs that have been approved or investigated in mUC, such as sacituzumab govitecan (SG) and trastuzumab deruxtecan (T-Dxd), lack CNS outcomes data in mUC but have initial clinical evidence of intracranial activity in breast cancer.^13–17^ However, it is difficult to generalize these outcomes to another tumor type or to an ADC with a different target and payload.^17,18^

The incidence of CNS metastases across cancer types, including mUC, is increasing, likely due to the increased efficacy of new treatments that prolong survival and extend the time period when CNS metastases can occur.^19^ Significant recent advances in the mUC treatment landscape warrant a reevaluation of the incidence and management of CNS metastases in this patient population.^20^ In this case report, we present our institutional experience of patients with mUC who experienced CNS progression either during or following EV treatment.

## Results

We identified 67 patients with mUC who were treated with EV at our institution between November 2017 and March 2023. In this cohort, the median age was 69, 51 (76%) patients were male, and 41 (61%) had pure urothelial carcinoma. The primary tumor site was bladder for 43 (64%) patients, upper tract for 15 (22%), and other/unknown for 9 (14%). Within this cohort, we identified 9 patients who experienced progression in the CNS during or after treatment with EV (Table 1). The median age was 59, 7 (78%) patients were male, and 6 (67%) had pure urothelial carcinoma. Primary tumor site was bladder for 5 (56%) patients, upper tract for 2 (22%), and other/unknown for 2 (22%). Among these patients, 6 had metastases in the brain parenchyma, 1 in the parenchyma and dura mater, 1 in the dura mater alone, and 1 in the leptomeninges alone. Two patients had CNS-only progression in the absence of any other systemic progression. The overall best response to EV as well as determination of progression in the CNS were determined by the investigator based on available clinical and radiographic data. Four patients received EV monotherapy, and 5 received EV as part of a combination regimen. At the time of CNS progression, 4 patients were on active EV treatment (within 6 weeks of last EV dose), 2 had discontinued EV and were not receiving any systemic therapy, and 3 patients were receiving subsequent therapy after progression on EV. For 5 patients not on active EV treatment, median time from EV discontinuation to CNS progression was 4.5 months (range: 3–18 months).

### Case 1

A 59-year-old man with muscle-invasive bladder cancer (MIBC) with urothelial histology was treated with neoadjuvant dose-dense methotrexate, vinblastine, doxorubicin, and cisplatin (ddMVAC) and radical cystectomy, but then had metastatic recurrence in the lungs. Combination pembrolizumab and *FGFR3* inhibitor therapy was initiated. After progression, he started EV with a partial response (PR) after 2 cycles (8 weeks) and a complete response (CR) after 5 cycles (21 weeks). During cycle 8 (34 weeks), he reported a new headache; MRI brain revealed leptomeningeal enhancement suggestive of carcinomatosis. There was no other systemic disease recurrence noted. His cognitive status declined progressively over several days and he passed away within the week.

### Case 2

**Figure 1:**
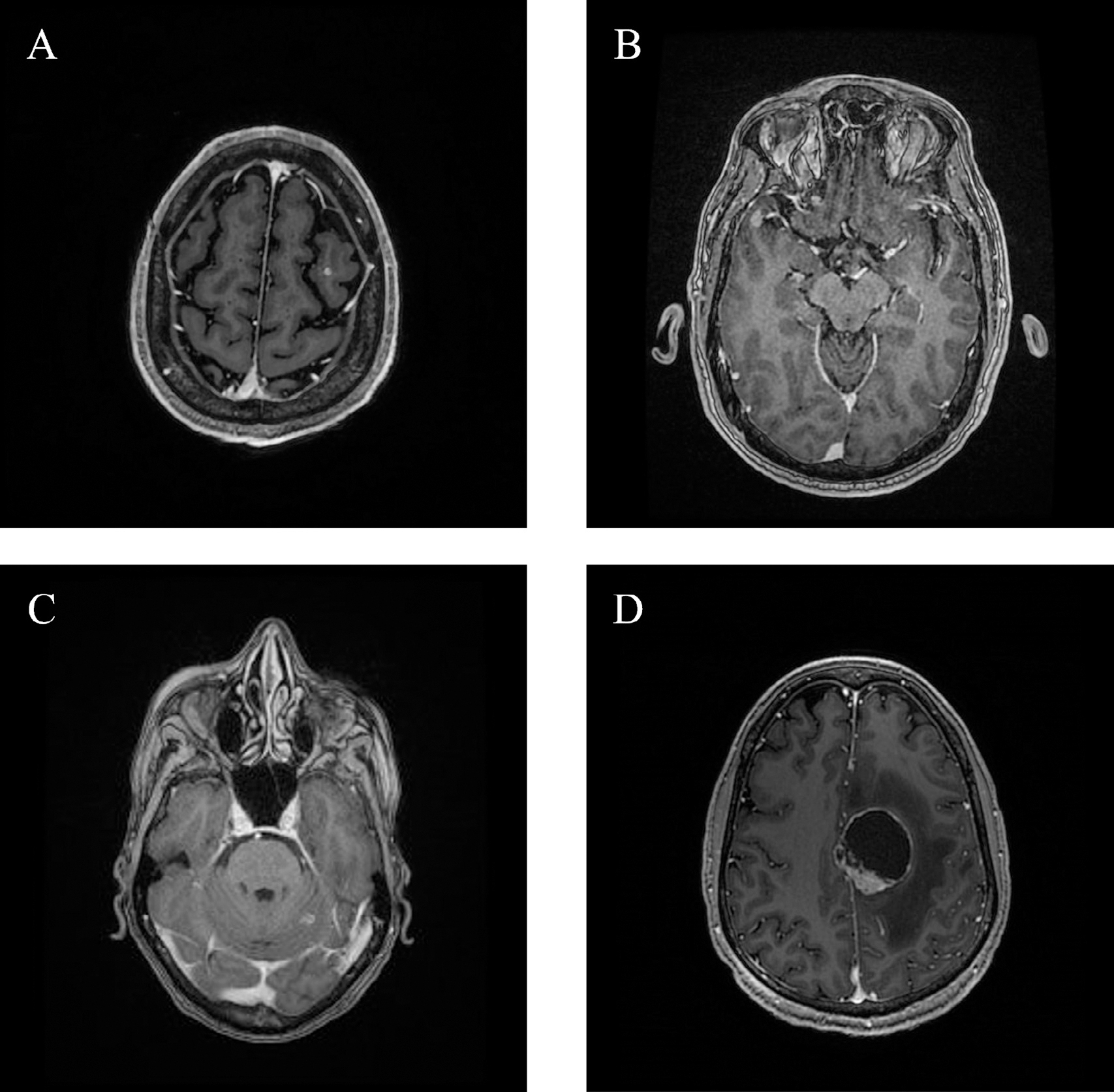
Representative CNS metastases from several patient cases (A) Case 2: Punctate enhancing lesion in the left middle frontal gyrus (B) Case 4: Hyperintense and enhancing focus in the right superior temporal gyrus (C) Case 6: Subcentimeter enhancing lesion in the superior left cerebellum (D) Case 7: Hemorrhagic cystic and solid lesion in the left cingulate gyrus and paracentral lobule.

A 52-year-old man presented with mUC and was started on EV after progressing on front-line cisplatin/gemcitabine chemotherapy. He received 2 cycles (8 weeks) of EV before restaging scans revealed progression in the right adrenal gland and liver. He also experienced headaches prompting a brain MRI, which revealed nonspecific enhancement in the superior parietal lobe and the occipital dura. EV was discontinued due to progression in the adrenal gland and liver, and he subsequently received 2 cycles of pembrolizumab before being hospitalized due to worsening fatigue and weakness. MRI brain revealed a new lesion in the left middle frontal gyrus suspicious for metastasis and dural thickening in the left occipital lobe suspicious for interval hemorrhage involving a dural metastasis (Figure 1). He passed away shortly thereafter.

### Case 3

A 56-year-old man with urothelial carcinoma underwent a nephroureterectomy and experienced metastatic recurrence initially treated with 6 cycles of gemcitabine/cisplatin followed by 2 cycles of maintenance avelumab. Upon disease progression, he initiated EV and had a CR after 1 cycle (4 weeks). EV was dose reduced to 1 mg/kg starting in cycle 2 and held after the first dose of cycle 3 due to progressive treatment-related neuropathy. CT scans then showed disease progression in the lymph nodes, and he received radiation therapy (RT) to supraclavicular and cervical chain lymph nodes followed by 3 cycles of pembrolizumab. Subsequent PET-CT showed further progression systemically and suspected intracranial metastases. MRI brain confirmed 2 partially hemorrhagic enhancing masses in the right cerebellar hemisphere and left parietal lobe. He received whole brain RT (WBRT) and, due to prior treatment-related neuropathy, continued EV at a reduced dose of 0.75 mg/kg. He remained on EV at the time of review.

### Case 4

A 61-year-old man with plasmacytoid bladder cancer underwent a cystoprostatectomy followed by adjuvant cisplatin/gemcitabine and, upon metastatic progression, 3 cycles of pembrolizumab. After progression, he received 4 cycles of EV (16 weeks) before subsequently progressing with new osseous and liver lesions. He then experienced monocular blurry vision; MRI brain revealed a lesion in the superior temporal gyrus and optic nerve enhancement (Figure 1). He continued to decline clinically without further treatment and subsequently passed away.

### Case 5

A 59-year-old man who presented with mUC, underwent a palliative cystoprostatectomy for bladder cancer and a concurrent total hip arthroplasty for an acetabular metastasis; then was treated with a combination of cisplatin, EV, and pembrolizumab as part of a clinical trial. He obtained a baseline MRI brain without contrast, which showed no evidence of metastasis. Initial restaging scans demonstrated stable disease (SD), but cisplatin was discontinued following 3 cycles (9 weeks) due to hearing loss. EV and pembrolizumab were later discontinued after 7 total cycles (21 weeks) due to progression in pelvic and osseous lesions. He subsequently received 9 cycles of gemcitabine/carboplatin. Following progression, he was briefly treated on a clinical trial of an immunotherapy combination and then received 9 cycles of SG but had progressive disease. After an additional 1 cycle of docetaxel, he experienced leg weakness and slurred speech. MRI brain revealed innumerable supra- and infratentorial metastases. He received WBRT but clinically progressed and transitioned to hospice.

### Case 6

A 72-year-old woman with a prior radical cystectomy for urothelial carcinoma of the bladder experienced metastatic recurrence in the liver and initiated a combination of cisplatin, EV, and pembrolizumab as part of a clinical trial. After completing 6 cycles (21 weeks) of cisplatin, she had a CR and continued EV and pembrolizumab for a total of 17 cycles (59 weeks) before being hospitalized for acute renal failure. Systemic treatment was discontinued. Three months later, she was hospitalized with seizures; MRI brain revealed metastases in the right superior frontal lobe and superior left cerebellum (Figure 1). She received WBRT but continued to decline clinically and transitioned to hospice.

### Case 7

A 36-year-old man underwent a nephroureterectomy for upper tract urothelial carcinoma but experienced metastatic recurrence in the lungs. He progressed sequentially on cisplatin/gemcitabine followed by erdafitinib. He then completed 2 cycles of EV (8 weeks) before starting combination pembrolizumab and EV, with pembrolizumab provided through a compassionate use program. After 1 cycle of combination therapy, he was hospitalized for sudden onset right leg weakness. MRI brain revealed multiple intracranial parenchymal lesions concerning for metastasis (Figure 1). He underwent metastasectomy in the left cingulate, which confirmed urothelial carcinoma, and received WBRT. He then restarted systemic treatment with combination EV/pembrolizumab. Subsequent scans revealed worsening systemic and intracranial metastatic disease. He was then switched to sacituzumab govitecan and received 3 cycles but had continued intracranial progression. He transitioned to hospice and subsequently passed away.

### Case 8

A 58-year-old woman presented with micropapillary mUC and initiated EV after progression on cisplatin/gemcitabine and avelumab switch-maintenance. After 1 cycle of EV, pembrolizumab was added and EV/pembrolizumab was continued for 18 months with PR as best response. EV was then stopped due to neuropathy and, 2 months later, pembrolizumab was also discontinued. After 2 months without treatment, she was hospitalized with altered mental status and possible seizure, leading to MRI brain revealing multiple supratentorial and infratentorial brain metastases. No other sites of progression were noted. She had WBRT and started T-Dxd given *ERBB2* alteration. At the time of review, the patient had a favorable radiographic response to radiation treatment and systemic therapy is ongoing.

### Case 9

A 72-year-old man with metastatic urethral squamous cell carcinoma was treated in sequence with TIP (paclitaxel, ifosfamide, cisplatin), olaparib (due to a *BRCA2* alteration), durvalumab/tremelimumab, and cisplatin/gemcitabine. Upon progression on the last regimen, he initiated EV, and pembrolizumab was added after 1 cycle (3 weeks). EV/pembrolizumab was discontinued after 5 cycles (13 weeks) due to progression in the lung and lymph nodes. He was subsequently treated with an investigational trophoblast cell surface antigen 2 (TROP-2) ADC but experienced disease progression. He experienced seizures 3 months later, and an MRI brain revealed a dural metastasis. Metastasectomy was performed in the right temporal lobe and he restarted olaparib, but then passed away 6 months later.

## Discussion

This case series highlights CNS metastasis as a notable pattern of progression in patients with mUC during or following EV treatment. These 9 cases occurred among a total of 67 patients treated with EV at our institution, corresponding to a 13.4% incidence of CNS metastasis. The reported incidence of CNS metastasis in mUC ranges from 1% to 3% in prior literature, though this describes patients undergoing treatment before EV was routinely used. Our reported incidence in an EV-treated population is substantially higher than these prior reports.^2–5^ One possible explanation is the potential longer survival of patients with mUC in the era of novel therapies including immune checkpoint inhibitors and ADCs, resulting in more time when CNS metastases could develop. An alternative explanation is limited CNS activity for EV-based regimens, leading to the CNS becoming a sanctuary site for possible metastases. We note that the overall cohort of patients who received EV may not be representative of the broader population of patients with mUC as it is enriched for patients who have survived long enough to receive an EV-based regimen, particularly in a time when it was only approved in the treatment-refractory setting. Nonetheless, the higher than historical rate of CNS progression in this case series suggests that the pattern of disease progression in the CNS may be a critical part of the natural history of mUC progression in patients receiving novel treatments such as EV.

In this case series, all patients had next-generation sequencing (NGS) of tissue from either primary tumor or metastatic sites. The most common genetic alterations were *TP53* (6), *TERT* (5), *CDKN2A, RB1*, and *ERBB2* (4 each), which are commonly seen in UC.^21,22^ *FGFR3* alterations were present in 2 patients. Given the limited sample size in this case series, future studies in larger cohorts will be critical to identify associations between genomic correlates and CNS metastases.

The activity of EV in the CNS remains poorly characterized compared to other ADCs such as SG and T-Dxd, the latter of which has demonstrated intracranial responses in the TUXEDO-1 and DEBBRAH trials.^13–17^ In contrast, preclinical models showed limited CNS distribution of EV’s payload, monomethyl auristatin E.^23^ Moreover, membranous Nectin-4 protein expression is frequently decreased in metastatic tissue^24^; if true among CNS metastases in our cohort, this could theoretically limit EV activity. Interestingly, there have been case reports of intracranial responses in mUC patients with CNS metastases treated with EV.^12^ An important distinction is that our cohort included only patients who experienced CNS progression after initiating EV. Therefore, the CNS activity of EV remains unclear, which warrants further studies in pre-clinical models and larger patient cohorts.

Any potential relationship between EV treatment and CNS progression will likely take on increasing clinical significance as EV moves into earlier lines of treatment for mUC and MIBC.^25,26^ Although CNS imaging is not currently part of the standard disease monitoring for patients with mUC, this approach may have to be reconsidered as additional data and experience with EV are acquired in earlier treatment settings.

Limitations of this series include its small sample size, retrospective analysis, and lack of comparator controls. The cases described are heterogeneous in clinical characteristics, treatment history, and EV regimen, which limits conclusions regarding the relationship between EV treatment and CNS progression. Moreover, conclusions on the incidence of new-onset CNS metastasis are limited by the lack of routine baseline neuroimaging for mUC patients at EV start, which could result in asymptomatic CNS metastases occurring either before or after starting EV not being detected. Nevertheless, these hypothesis-generating findings suggest that the epidemiology of CNS metastases in urothelial cancer and the activity of EV in the CNS should be further explored in larger, more contemporary patient cohorts.

## Conclusion

This case series presents patients with mUC who progressed with CNS metastasis during or after treatment with EV. The incidence of CNS metastasis in patients previously treated with EV was 13.4% – higher than previously reported in mUC cohorts – highlighting the diagnosis and management of CNS metastases as emerging unmet needs in this patient population. Understanding the relationship between EV treatment and CNS metastases in this patient population will require larger, prospective studies.

## Figures and Tables

**Table 1:** Patient Characteristics in the Case Series

Pt.	Age at EV Start and Gender	Primary Histology	Systemic Therapies Prior to CNS Progression (In Addition to EV)	Time From Met Disease to EV Start (Months)	EV Regimen	Best Response to EV	Time on EV (Months)	Time From EV Start to CNS Metastases (Months)	Location of CNS Metastases	Patient Disposition	Pathogenic Genetic Alterations and HER2 Immuno-histochemistry (IHC), (if available)	Anatomic Site for Genomic Analysis (Sequencing Panel)
1	59-year-old man	Urothelial carcinoma	Neoadjuvant ddMVAC, Combination Pembrolizumab and Investigational FGFR3 Inhibitor	8.1	EV	CR	7.4	8.1	Leptomeninges	Hospice 11 days after EV discontinuation	*ARID1A*^e^, *FGFR1*^e^, *KDM6A*^e^, *MCL1*^e^, *NSD3*^e^, *RB1*^e^, *ROS1*^e^, *TERT*^e^, *TP53*^e^, *VHL*^e^, *ZNF703*^e^	Bladder (Foundation One CDx)
2	52-year-old man	Urothelial carcinoma	Cisplatin/Gemcitabine	6.4	EV	PD	1.8	3.2	Brain, Dura Mater	Hospice 23 days after discovery of brain metastases	*KMT2B*^e^, *TP53*^e^, *RB1*^e^, *TERT*^e^, *ERCC2*^e^, *HRAS*^e^, *PIK3CA*^e^ (IHC 0)	Bone (UCSF500)
3	56-year-old man	Urothelial carcinoma	Cisplatin/Gemcitabine, Maintenance Avelumab	8.8	EV	CR	2.2	12.5	Brain	Restarted EV treatment	*ARID1A*^e^, *ERBB2*^e^, *MYCL1*^e^, *RB1*^e^, *SRC*^e^, *TERT*^e^, *TP53*^e^	Lymph Node (FoundationOne CDx)
4	61-year-old man	Plasmacytoid variant with signet ring features	Adjuvant Cisplatin/Gemcitabine, Pembrolizumab	3.2	EV	SD	3.6	4.7	Brain	Deceased 34 days after discovery of brain metastases	*CDH1*^e^, *CTNNB1*^e^, *PTEN*^e^, *SMAD4*^e^, *TERT*^e^, *TP53*^e^	Ureter (FoundationOne CDx)
5	59-year-old man	Urothelial carcinoma	No Prior Systemic Therapies	3.7	EV / Pembrolizumab / Cisplatin	SD	4.8	23.1	Brain	Hospice 14 days after discovery of brain metastases	*PTCH1*^e^, *RBM10*^e^, *MRE11A*^e^, *TET2*^e^, *ERBB2*^e^, *TP53*^e^, *ARID1A*^e^, *CDKN2A*^e^, *CDKN2B*^e^ (IHC 1+)	Bladder (UCSF500)
6	72-year-old woman	Urothelial carcinoma	No Prior Systemic Therapies	2.1	EV / Pembrolizumab / Cisplatin	CR	13.6	17.0	Brain	Hospice within 3 weeks of brain met discovery	*ERBB2*^e^, *FGFR3*^e^, *BAP1^e^, CDKN2A*^e^, *CDKN2B*^e^, *TSC1*^e^ (IHC 3+)	Liver (UCSF500)
7	36-year-old man	Urothelial carcinoma	Cisplatin/Gemcitabine, Erdafitinib	13.7	EV / Pembrolizumab	SD	4.7	2.5	Brain	Started subsequent treatment but later progressed and passed away	*TERT*^e^, *CDKN1A*^e^, *TSC1*^e^, *CDKN2A/CDKN2B*^e^, *CCND1/FGF19/FGF4/FGF3*^b^ (Kidney/Ureter Only), *MYC* (Kidney/Ureter Only), *FGFR3-TACC3*^d^ (Lung and Brain Only) (IHC 2+ in lung, IHC 1+ in upper tract)	Kidney/Ureter, Lung, Brain (UCSF500)
8	58-year-old woman	Micropapillary urothelial carcinoma	Cisplatin/Gemcitabine, Maintenance Avelumab	8.0	EV / Pembrolizumab	PR	18.2	22.8	Brain	Started subsequent treatment	*ERBB2*^e^, *CCND1*^e^, *CDKN2A*^e^	Liver (Strata)
9	72-year-old man	Squamous cell carcinoma	TIP, Olaparib, Durval- umab/Tremelimumab, Cisplatin/Gemcitabine	32.0	EV / Pembrolizumab	PD	3.7	7.8	Dural	Deceased within 6 months	*NFE2L2*^e^, *TP53*^e^, *RB1*^e,a^	Lung (UCSF500)

a BRCA2 reversion occurred after treatment with olaparib and before treatment with EV.

b Amplification.

c Deep deletion.

d Fusion.

e Other alteration.

## Clinical Practice Points

- The incidence of CNS metastases in patients with mUC either during or following treatment with enfortumab vedotin may be higher than previously reported in similar mUC cohorts.
- CNS metastases in EV-treated patients with mUC are associated with inferior outcomes and limited systemic treatment options.
- Poor outcomes associated with CNS metastases highlight the need for greater vigilance regarding CNS progression in the population of mUC patients treated with enfortumab vedotin and a reevaluation of the role of CNS imaging surveillance in this disease state.
